# Physicochemical Properties of Nucleoli in Live Cells Analyzed by Label-Free Optical Diffraction Tomography

**DOI:** 10.3390/cells8070699

**Published:** 2019-07-10

**Authors:** Tae-Keun Kim, Byong-Wook Lee, Fumihiko Fujii, Jun Ki Kim, Chan-Gi Pack

**Affiliations:** 1 Asan Institute for Life Sciences, Asan Medical Center, Seoul 05505, Korea; 2 Division of Physical Pharmacy, Faculty of Pharmaceutical Sciences, Kobe Gakuin University, Kobe 650-8586, Japan; 3 Department of Convergence Medicine, University of Ulsan College of Medicine, Seoul 05505, Korea

**Keywords:** optical diffraction tomography, fluorescence correlation spectroscopy, nucleolus, refractive index, volume, diffusion coefficient, liquid-liquid phase separation

## Abstract

The cell nucleus is three-dimensionally and dynamically organized by nuclear components with high molecular density, such as chromatin and nuclear bodies. The structure and functions of these components are represented by the diffusion and interaction of related factors. Recent studies suggest that the nucleolus can be assessed using various protein probes, as the probes are highly mobile in this organelle, although it is known that they have a densely packed structure. However, physicochemical properties of the nucleolus itself, such as molecular density and volume when cellular conditions are changed, are not yet fully understood. In this study, physical parameters such as the refractive index (RI) and volume of the nucleoli in addition to the diffusion coefficient (*D*) of fluorescent probe protein inside the nucleolus are quantified and compared by combining label-free optical diffraction tomography (ODT) with confocal laser scanning microscopy (CLSM)-based fluorescence correlation spectroscopy (FCS). 3D evaluation of RI values and corresponding RI images of nucleoli in live HeLa cells successfully demonstrated varying various physiological conditions. Our complimentary method suggests that physical property of the nucleolus in live cell is sensitive to ATP depletion and transcriptional inhibition, while it is insensitive to hyper osmotic pressure when compared with the cytoplasm and nucleoplasm. The result demonstrates that the nucleolus has unique physicochemical properties when compared with other cellular components.

## 1. Introduction

The cell nucleus is three-dimensionally organized into subnuclear structures, including chromatin containing genomic information and nuclear bodies such as the nucleolus, nuclear speckles, and Cajal bodies. Among them, the nucleolus, a factory for ribosome biogenesis, is considered as related to various cell functions such as aging, tumor suppressor regulation, and stress sensing [1,2,3]. It has been demonstrated that the nucleolus contains high concentrations of over 489 proteins, which vary in function and cellular condition [4]. Additionally, the organization and function of the nucleolus is maintained by high mobility and interaction kinetics of nuclear and nucleolar factors and ribosomal DNA/RNA (rDNA/RNA) [5,6,7], although the nucleolus shows a densely packed structure in fixed cells observed by electron microscopy. However, many studies have demonstrated that the nucleoli are dynamic structures with highly mobile constituents that can diffuse in and out of the nucleoplasm. Recently, a physicochemical phenomenon known as liquid–liquid phase separation (LLPS) has been increasingly recognized as an important factor in the formation, maintenance, and function of the subnucleolar structures known as the tripartite structure which includes the fibrillar center (FC), dense fibrillary component (DFC), and granular component (GC) [8,9,10,11,12,13]. LLPS, which is accomplished by weak interactions among related components and reversible chemical processes, is consistent with the concept of self-organization originated from dynamic properties of nuclear structures such as nuclear bodies and transient interactions of their components [6,14]. Advances in live cell imaging based on fluorescence microscopic techniques have provided insight into the dynamic properties of nuclear organelles and their components such as nuclear proteins. Previous studies using biophysical methods such as fluorescence recovery after photobleaching (FRAP) and fluorescence correlation spectroscopy (FCS) have demonstrated that molecular diffusion and shuttling and the interaction kinetics of nuclear proteins contribute to the organization and function of the nucleolus [5,15,16,17,18,19]. These studies indicate that molecular dynamics are physiologically important and that the nucleolus is not a static rigid structure, but rather a highly flexible structure [14]. Moreover, studies using probe GFPs have shown that probes of various sizes are accessible and freely diffuse in various nuclear and mitotic compartments, such as chromosomes and nucleoli of live cells [20,21,22], suggesting that the compartments have a fluidic property.

The cellular microenvironment comprises key physicochemical characteristics of various cellular compartments with a high macromolecular content [23,24,25,26,27]. Previous studies using confocal microscopy, FCS, and single-molecule imaging have demonstrated that condensed chromatin domains and mitotic chromosomes are freely accessible to large macromolecules, which has important implications for understanding large-scale chromatin and chromosome structure [28,29]. Because the diffusion coefficients (Ds) of probe molecules in live cells as well as in aqueous solution provide useful information regarding the fluidic and unique microenvironment such as the molecular crowding effect [30,31,32,33,34], the *D* value of biologically inert probes such as multimeric GFPs have been systematically characterized to investigate these traits [21,28,30,31,32,33,34]. Studies have demonstrated that the local viscosity and accessibility of nuclear compartments such as the nucleolus, nucleoplasm, and mitotic chromosome can be differentiated and characterized based on the *D* value of the probe molecule detected in the compartments [20,21,22,28]. Particularly, studies using FCS indicated that the nucleolus and mitotic chromosomes of cells are accessible to protein factors with molecular sizes of up to 150 kDa (i.e., pentameric GFP), despite their highly dense structures [21,28], although the differences in *D* values in the three subnucleolar compartments has not been evaluated in detail. Moreover, previous studies suggested that the local viscosity of the nucleolus was 10-fold higher than that of water for monomer GFP and that the nucleolar viscosity experienced by the probe molecule was significantly dependent on the size of the probe molecule [20,21]. These results suggest that subnucleolar compartments are susceptible to molecular crowding effects that occur in the nucleolus compared to in the nucleoplasm [35]. Similarly, mitotic chromosomes were found to be accessible by multimeric GFP molecules and the local viscosity of chromosomes was much lower than that of the nucleolus, although chromosomes also have highly dense structure [28]. By using the experimental *D* values and molecular simulation based on the *D* values, the accessibility and high mobility of probe molecules in mitotic chromosomes can be explained by local nucleosome fluctuation (i.e., constrained diffusion of the nucleosome itself). The *D* value of GFP probes in the nucleolus is much smaller than that in the mitotic chromosome; thus, the molecular density of the nucleolus, which shows increased fluidic viscosity, may be much higher than that of the mitotic chromosome. Otherwise, the mobility of nucleolar components or large ribonucleoprotein particles is much slower than that of the nucleosome, although the effect of mobility of nucleolar factors such as nucleolar proteins and ribonucleoprotein particles on *D* value of probes has not been demonstrated.

Accordingly, the physicochemical properties of the nucleolus and other dense cellular compartments under various physiological conditions remain unclear. Fluorescence methods used in previous studies have only traced fluorescently labeled target or probe molecules under various physiological conditions. Because molecular dynamics and interactions in cellular compartments are generally related to structural or physicochemical changes in the surrounding microenvironment (i.e., LLPS and molecular crowding effect), quantifying the overall physicochemical properties of the nucleolus and dynamic properties of target/probe molecules is helpful for understanding nucleolar organization and function under various conditions.

Although FCS is highly sensitive and well suited to measuring the diffusional mobility of probe molecules in very small regions that comprise subnuclear compartments such as the nucleolus, FCS measurements of whole-nucleolar areas are time-consuming and too inefficient to allow mobility distribution. Moreover, the phototoxic and bleaching effects of fluorescence methods such as confocal microscopy and FCS must be also carefully considered when attempting to obtain reliable information from live cells, especially stress sensing compartments.

In practice, fluorescence methods of CLSM and FCS have limitations such as phototoxicity, long scanning time for 3D imaging, and time-consuming measurements. On the contrary, the label-free quantitative phase imaging (QPI) method has no specificity of target molecules and cannot distinguish between different cellular components if there are no differences in molecular density. Nevertheless, several label-free QPI methods such as optical diffraction tomography (ODT) were recently demonstrated as promising methods for high-speed and 3D live cell imaging [36,37,38,39,40]. ODT is an interferometric microcopy technique that acquires 3D RI tomograms of cells without fluorescent labeling. ODT measurement is fast and can acquire one 3D RI tomogram in <1 s [41]. Optically transparent cells without fluorescent tags are excited by low light intensities, and ODT minimizes the phototoxicity on the cells, making it suitable for noninvasive measurement of live cells. Importantly, ODT provides absolute parameters such as volume and refractive index (RI) [42]. Since the RI is generally proportional to the molecular concentration [43], which is related to the viscosity of solutions, FCS and ODT analysis on live cells will be complementary.

In the present study, we examined the application of our method for the quantification of several physical parameters of the nucleolus, including volume, RI, and local viscosity (i.e., *D* value). Although there was a limitation for nucleolar volume evaluation under various physiological conditions, we successfully acquired 3D RI images of the nucleolus of live HeLa cells and quantified the characteristic parameters by combined CLSM and FCS analyses. The label-free RI tomographic images of nucleoli suggest that the nucleolus could be described by multiple bands with different RI ranges as well as by a single broad-range RI band. Moreover, we quantitatively compared the RI values of the nucleolus, nucleolar volume, and *D* value of GFP probe inside the nucleolus under various physiological conditions, finding that the nucleolus has unique physical properties different from other cellular compartments.

## 2. Materials and Methods

### 2.1. Cell Culture and Treatments

HeLa cells were cultured at 37 °C in 5% CO_2_ in DMEM supplemented with 10% FBS, 100 U/mL penicillin, and 100 U/mL streptomycin. For live cell microscopic analysis, HeLa cells were plated and cultured in tomo-dishes (Tomcube, Korea) for ODT observation or Lab-Tek 8-well chambered coverglass (Nunc, USA) for CLSM and FCS observations. Plasmid constructs of pmRFP-C1-B23 and pmRFP-C1-fibrillarin were obtained as gifts from Dr. T. Saiwaki (Osaka University, Osaka, Japan) and used to express nucleophosmin (RFP-B23) or fibrillarin (RFP-Fib) tagged with momomeric RFP [15,21]. Plasmid constructs of pEGFP_5_-C1 (pentameric EGFP) were used to express pentameric GFP (GFP_5_) [20,21]. The constructs were verified by sequencing and purified using a plasmid DNA midiprep kit (QIAGEN, Hilden, Germany). For observation of HeLa cells expressing mRFP-B23 or GFP_5_ in CLSM and FCS, cells were transiently transfected using Lipofectamine 3000 (Thermo Fisher Scientific, USA) as per manufacturer instructions. To investigate the influence of different osmotic conditions, HeLa cells were incubated with either Dulbecco’s PBS (Biowest, France) for hypotonic conditions, or phenol-free DMEM supplemented with 10% FBS and 0.2 M sucrose for hypertonic condition. PBS or phenol-free DMEM supplemented with 10% FBS and 0.2 M sucrose was applied prior to imaging. To investigate the influence of ATP depletion, fresh DMEM without glucose was added before treatment, and 6 mM 2-deoxyglucose (2-DG) and 10 mM NaN3 (sodium azide) were used. For transcriptional inhibition of pol I, II, and III, fresh media were added before treatment, and then high concentration, 4 μg/mL, actinomycin D (Act D, Sigma) was treated. The osmotic concentration of the media was measured with an osmometer (Fiske Micro-Osmometer Model210, Advanced Instruments, Norwood, MA, USA). The RI value of the culture media was measured using a refractometer (Abbemat 3200; Anton Paar GmbH, Graz, Austria) and used as the reference RI value for ODT measurement of cells.

### 2.2. Optical Diffraction Tomography (ODT)

ODT reconstructs the 3D RI tomogram of a sample from multiple 2D images of the sample acquired at various illumination angles, which is similar to a well-known computed tomography method for measuring X-ray absorptivity [44]. ODT measurements were performed at 37 °C using a commercial ODT microscope with excitation at 532 nm (HT-2H, Tomocube, Inc., Daejeon, Korea) as described previously [41,44,45]. Briefly, a Mach–Zehnder interferometric microscope was used to reconstruct the cell 3D RI tomograms. The commercial microscope consists of an illumination/sample modulation unit and an optical field recording unit. Optical interference was used to capture amplitude and phase information from light transiting a cell sample. The measured RI value of each medium by the refractometer was used as a reference for ODT cell measurement. To verify the capability of our set up, polystyrene beads with a diameter of 3 μm (Sigma-Aldrich, St. Louis, MO, USA) were used [45]. The lateral and axial optical resolutions of our ODT system were 110 and 356 nm, respectively. The resolution of the RI values was less than 0.001 [44].

### 2.3. Confocal Laser Scanning Microscopy (CLSM)

Fluorescence microscopy using live cells was performed using an inverted confocal laser scanning microscope (LSM780; Carl Zeiss, Germany). For 3D and time-lapse images, HeLa cells expressing RFP-B23 were excited at a wavelength of 561 nm, and the emission signal band was detected at 570–630 nm. The interval for time-lapse imaging was 3 min, and z-stack images were taken at 0.5 µm intervals using a 5× magnification objective lens (C-Apochromat, 63×/1.2NA). All live cell measurements were performed at 37 °C in 5% CO_2_ culture conditions. Fluorescence images were processed using software (Zen 2012 SP5; Carl Zeiss, Germany) installed on an LSM780 confocal microscope system for 3D reconstruction or intensity profile analyses.

### 2.4. Fluorescence Correlation Spectroscopy (FCS)

Confocal imaging for FCS measurement was performed using an LSM780 confocal microscope system (Carl Zeiss, Germany). GFP was excited at 488 nm using a CW Ar^+^ laser through a water-immersion objective (C-Apochromat, 40×, 1.2 NA; Carl Zeiss). All CLSM observations and corresponding FCS measurements were performed at 25 °C as described previously [20,21]. Briefly, GFP was excited with minimized power at 488 nm with an argon ion laser through a water-immersion objective (C-Apochromat 40×/1.2NA; Carl Zeiss). All fluorescence autocorrelation functions (FAFs) were measured for 10 s at least five times with 2 s intervals to exclude nonstationary fluorescent fluctuations due to slow *z*-axis drift of cells and to slow the photobleaching effect over the measurement duration.

Data analysis for FAFs was performed as previously described [20,21]. To obtain the diffusion time, FAFs [*G* (τ)] of the measurements were fitted using the following one-component model with or without a triplet term:

(1) Gτ=1+1N∑iyi11+ττDi11+1s2ττDi12

where *N* is the number of molecules in the detection volume, τ*_Di_* is the correlation time, *w* and *z* are width and axial length of the detection volume, respectively, and *s* is the structure parameter *z*/*w*. Note that the triplet term is not shown in the equation for simplicity, since the triplet is an independent term with diffusional mobility. Diffusion times had the following relationship to the diffusion coefficient:

(2) $$\tau_{D_{i}}=\frac{w^{2}}{4D_{i}}.$$

The diffusion coefficient of pentameric GFP (*D*_GFP_) was calculated from the reported value of the diffusion coefficient of rhodamine 6G (*D*_Rh6G_ = 280 μm^2^/s), and the corresponding measured diffusion times of Rh6G (*τ*_Rh6G_) and GFP (*τ*_GFP_) were given as follows:

(3) $$\frac{D_{GFP}}{D_{Rh6G}}=\frac{\tau_{Rh6G}}{\tau_{GFP}},$$

All FAF curves from FCS measurements were fitted using the fitting program installed on the LSM780 system with the model equation (1) as demonstrated previously [20,21]. The FAFs in cells were fitted by a 1- or 2-component model (*i* = 1 or 2) to consider two different types of diffusion. The diffusion coefficient obtained from the 1-component model was represented by *D*. For the nucleolus, a proportion of FAFs were fitted by a 2-component model analysis. Larger *D* values from the 2-component model analysis, which correspond to the *D* values in the nucleoplasm (i.e., fast diffusion component), were excluded for analytical simplicity. Instead, the smaller *D* values (i.e., slow diffusion component) were considered characteristic values representing the fluidic property of the nucleolus [21].

### 2.5. Image Rendering, RI, and Volume Calculation

The RI isosurfaces of HeLa cells were rendered and the RI values was evaluated using a software installed on an ODT instrument (TomoStudio, Tomocube, Inc., Korea). In brief, user-defined transfer functions representing a range of the RI and the RI gradient magnitude were changed in the virtual palette in the software until the 3D-rendered images of the cellular compartments such as the nucleolus were sufficiently described compared with the raw ODT images. The 3D rendered images were compared with 3D images obtained from CLSM. For quantification, the median value of RI range for rendering was used for calculating mean RI values of the nucleolus and cytosol. For calculation of cell volume, a 3D-rendered image was used on a software from Tomocube Inc. (Chromosome analysis, Tomocube Inc., Korea). Fluorescent images of RFP-B23 taken using the confocal microscope were also 3D-rendered using the IMARIS 8.1.2 software (Bitplane, USA) and used for the nucleolar volume analysis. Where necessary, the surface function of the IMARIS software was used.

### 2.6. Statistical Methods

Student’s *t*-tests were performed for comparison of mean values to determine significance (Origin v.8.5, Northampton, MA, USA). All *p*-values less than 0.05 were considered significant.

## 3. Results and Discussions

### 3.1. Label-free RI 3D Images of the Nucleolus in Live HeLa Cells

**Figure 1:**
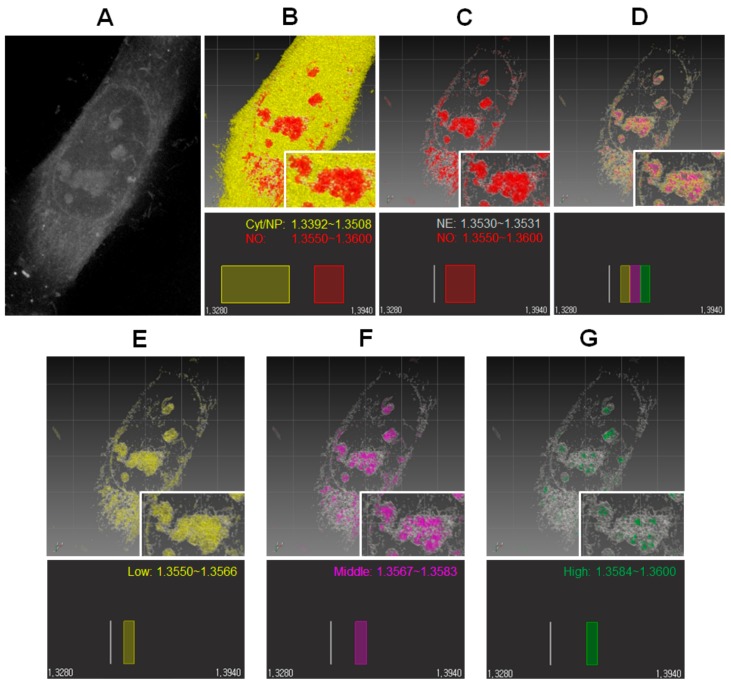
The nucleolus of a live HeLa cell imaged by label-free optical diffraction tomography (ODT). (**A**) Representative raw ODT image of a HeLa cell. (**B**) Pseudo-colored refractive index (RI) image of the cell. The cytoplasm and nucleoplasm are represented by yellow and the multiple nucleoli in the cell nucleus by red, respectively (upper). The RI bands used for pseudo-coloring the cell are shown (lower). (**C**) Pseudo-colored RI image of the same cell, highlighting nucleoli using a single broad RI band (lower). White color presents the nuclear membrane, the nucleoli boundary region, and also parts of an unknown structure in the perinuclear region of the cytosol. (**D**) A pseudo-colored RI image of the same cell, highlighting nucleoli with three narrow RI bands with identical widths (lower) is shown for comparison with C. (**E**–**G**) Pseudo-colored RI images of the nucleoli shown in D, but showing only single narrow RI bands, each with a different RI range (lower). Enlarged images of the largest nucleolus are shown for clarity (insets).

To demonstrate how RI images of the nucleolus were accurately constructed by ODT analysis, live HeLa cells were measured by ODT. Due to the molecular high density of the nucleolus, it was expected that tomographic RI images of the cells would have a clear nucleolar structure when compared with other nuclear and cytosolic compartments in S-phase. In practice, Figure 1A shows representative raw ODT image of a live HeLa cell. The cell presents multiple rounded components of high molecular density in the nucleus, the contour of the nuclear membrane, and the fibric structure in the cytosol. Since the nucleolus has the highest molecular density in the nucleus, the five rounded components were assumed to be nucleoli. For simplicity, other small components of high molecular density under 2 μm were not considered as nucleoli. Figure 1B,C shows two different pseudo-colored RI images of the identical cell shown in Figure 1A. Figure 1B presents the cell simply described by two RI bands of broad range (low and high). The low RI band fully fills both the cytosol and nucleus. The high RI band homogenously fills five rounded components (i.e., five nucleoli) in the nucleus (Video S1). Besides the five nucleoli, discontinuous nuclear membrane and fibric component in a perinuclear region of cytoplasm with high density were partially presented, since their RI falls within the high RI range. In contrast, Figure 1C shows the cell described by one narrow RI band and the high broad band used in Figure 1B. The narrow RI band presents a part of the nucleolar surface and fibric components in the cytosol as well as the rounded nuclear membrane, even though the identity of the surfaces and cytosolic components are not unclear. Since it is known that the nucleolus has heterogeneous subcompartments such as the fibrillar center (FC), dense fibrillary component (DFC), and the granular component (GC) with different molecular densities, the single broad RI bands shown in Figure 1B,C was divided by three narrow RI bands (low, middle, and high) and pseudo-colored RI images of the nucleolus were examined by the respective narrow bands (Figure 1D–G). The low RI band homogenously fills a broad range and outer part of the nucleolus, whereas the middle and high RI bands occupy inner heterogeneous volumes in the nucleolus. Interestingly, high RI bands occupy a much small volume surrounded by the region of middle RI bands (Video S2). Although the segmentation of one broad RI band into three bands is reasonable for properly expressing the heterogeneous molecular density of the nucleolus, what each volume of different RI corresponds to is not clear. Correlative or complementary analysis with fluorescent or electron microscopic methods will be helpful for uncovering such differences.

### 3.2. Comparison between RI and CLSM Images of the Nucleolus at Various Physiological Conditions

**Figure 2:**
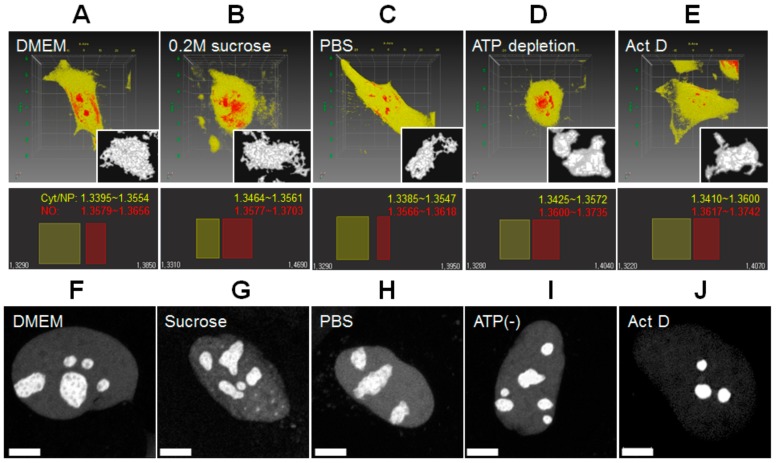
Comparison of 3D confocal fluorescence images of B23-mRFP with 3D RI images of the nucleolus of live HeLa cells under various physiological conditions. (**A**–**E**) Representative RI images of live HeLa cells under normal (DMEM, 330 mOsm/kg), hypertonic (0.2 M sucrose, 500 mOsm/kg), hypotonic (PBS, 290 mOsm/kg), ATP depleted, and Act D treatment conditions are shown (upper). For simplicity, both the cytoplasm and nucleoplasm are represented by a single RI band (yellow), while the multiple nucleoli in the nucleus are highlighted with a different RI band (red). For morphological clarity, an enlarged image of the largest nucleolus of each cell is shown (inset, gray). Each RI band and the range used for pseudo-coloring is indicated (lower). It should be noted that the same pseudo-coloring of the cytoplasm and nucleoplasm was used for clarity of cell morphology and comparison between the conditions. Scale square, 10 × 10 μm. (**F**–**J**) Representative confocal images of B23-RFP-expressing HeLa cells under normal (DMEM), hypertonic (0.2 M sucrose), hypotonic (PBS), ATP depleted, and Act D treatment conditions. Scale bar is 5 μm.

**Figure 3:**
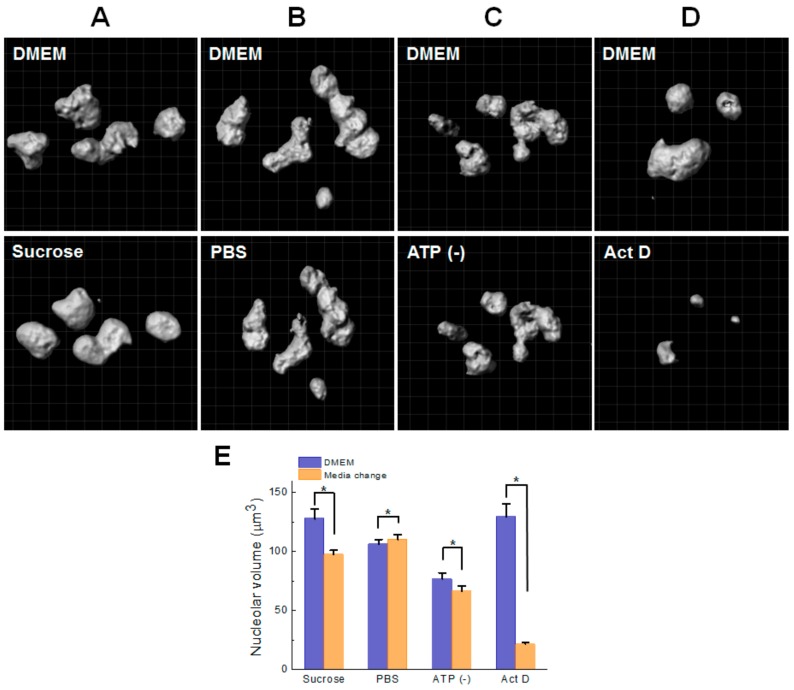
3D confocal image analysis of the nucleolus in single HeLa cells. (**A**–**D**) Representative 3D rendered confocal images of the multiple nucleoli in the nuclei of single HeLa cells expressing B23-mRFP before (DMEM media, upper) and after changes to hypertonic media (0.2 M sucrose, 10 min), hypotonic media (PBS, 10 min), ATP depletion (30 min), and Act D treatment (2 h) are shown respectively (lower). For evaluation of morphological changes, the volume and surface softness were processed by IMARIS software as described in *Materials and Methods*. (**E**) Mean nucleolar volume of single HeLa cells expressing B23-mRFP. The changes in volume of single cells were measured by CLSM under the physiological conditions described in A–D (mean ± SEM; n = 20 cells for each condition) * *p* < 0.05.

To investigate the influence of physiological changes on the structure and physicochemical properties of the nucleolus in live HeLa cells, the physiological state of cells was changed by varying osmotic pressure, ATP depletion, and transcriptional inhibition [20,25,46]. Since live HeLa cells are normally cultured with DMEM supplemented with 10% FBS (330 mOsm/kg), hypertonic conditions were prepared by adding 0.2 M sucrose (500 mOsm/kg), and relatively hypotonic conditions were prepared by adding PBS (290 mOsm/kg) [25]. Figure 2A–E shows the representative pseudo-colored RI images of live HeLa cells under different physiological conditions. It should be noted that a single RI band for the nucleolus and a single broad RI band of the cytoplasm and nucleoplasm with the same pseudo-color were used for structural clarity and comparison between the conditions. Instead, RI ranges corresponding to the cytoplasm, nucleoplasm, and the nucleolus are shown, respectively. All RI values of the cytoplasm, nucleoplasm, and the nucleolus increased under hypertonic condition, ATP depletion, and transcriptional inhibition, but decreased under hypotonic condition. The difference of RI values between the nucleolus and cytoplasm (nucleoplasm) under the conditions of ATP depletion and transcriptional inhibition were larger than that those under other conditions. Morphologically, 3D RI images of the nucleolus observed in normal, hypertonic, and hypotonic conditions presented rugged surface, whereas those observed in conditions of ATP depletion and transcriptional inhibition presented rounded and flat surface, suggesting that nucleolar structure was changed or reorganized. Moreover, size of the nucleolus of cells under transcriptional inhibition was much smaller than those under other conditions, which is in close agreement with previous studies [16,21,47]. In addition to ODT observation, HeLa cells expressing B23-RFP, as a maker protein for the nucleolus [47], were also observed by CLSM (Figure 2F–J). Fluorescent 2D images of B23-RFP observed in isotonic, hypertonic, and hypotonic conditions presented a punctate structure, whereas those observed in conditions of ATP depletion and transcriptional inhibition presented compact and evenly bright structure, suggesting that the localization of nucleolar B23-GFP was reorganized. Nucleolar reorganization by transcriptional inhibition (i.e., Act D treatment), such as segmentation (i.e., disruption) and size decrease of the nucleolus (i.e., release of ribosomal proteins), has been demonstrated by previous studies [1]. Our result suggests that the structural changes induced by ATP depletion and transcriptional inhibition observed by CLSM and ODT are correlated. Although ODT measurement provided volume of the nucleolus, nucleolar volume was not evaluated by ODT, but by fluorescent 3D image analysis. Since unknown components of high molecular density (i.e., high RI value) were induced around and in the nucleus under hypertonic condition and ATP depletion, it was difficult to exactly quantify nucleolar volume. Instead, we adopted 3D and time-lapsed CLSM analysis for evaluating volume change of the nucleolus in the identical single-cell varying conditions. Figure 3A–D shows a representative 3D-rendered CLSM image of B23-RFP in single cells varying the physiological condition. The rough surface of the nucleolus was changed into a smooth surface by hypertonic treatment. As expected, the volume of the nucleoli in normal culture media was largely decreased by transcriptional inhibition. On the contrary, media change from DMEM to PBS and ATP depletion showed no specific change of the nucleolar morphology. Figure 3E summarized the volume change of nucleoli in single HeLa cells estimated by CLSM 3D measurement. The mean volume of nucleoli was dramatically decreased by hypertonic treatment and transcriptional inhibition. A small increase and decrease by hypotonic treatment and ATP depletion, respectively, were observed. Notably, the mean volumes of cells cultured in DMEM differed in each single-cell experiment. It is likely that the differences originated from the heterogeneity of cells transfected with B23-mRFP in different phases of the cell cycle. This difference can be improved by performing synchronized culture of transfected cells or by using stably expressing cell lines. However, the results of single-cell observation clearly revealed changes in the nucleolar volume when the media was changed. Because high hypertonic stress enforces molecular crowding in cellular components because of the osmotic extraction of water from the cells [25], the nucleolar volume and cell volume were expected to decrease. In practice, ODT analysis showed that the cell volume was significantly changed by hypertonic stress (Figure S1). Under hypotonic conditions, it is likely that a small decrease (40 mOsm/kg) in osmolarity insignificantly induces influx of water into the cells. Although a previous study showed that ATP depletion induces nuclear shrinkage of live HeLa cells expressing H2B-YFP [46], it remains unclear if the nucleolar size is also changed by ATP depletion. Our results suggest that shrinkage of the nucleolar volume can be accompanied by nuclear shrinkage induced by ATP depletion (Figure S1). The dramatic decrease in the nucleolar volume induced by Act D treatment is consistent with previous studies demonstrating that rDNA transcriptional inhibition by inhibitors induces nucleolar segregation and decreased size [8,17,21].

### 3.3. Quantification of Biophysical Parameters of the Nucleolus in Live HeLa Cell

**Figure 4:**
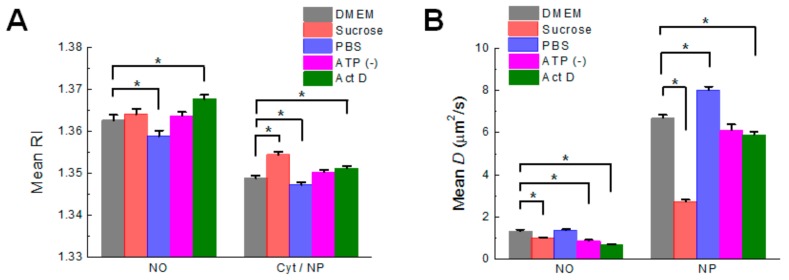
Evaluation of RI and volume of the nucleolus and diffusion coefficient *D* for GFP in the nucleolus under different physiological conditions. (**A**) Mean RI of the nucleolus and cytosol is shown. The RI of each compartment was measured by ODT under the five different conditions indicated (mean ± SEM; n = 20 cells). * *p* < 0.05. (**B**) Mean *D* value for pentameric GFP in the nucleolus of HeLa cells. *D* values of pentameric GFP were evaluated by FCS analysis using Equations (1), (2) and (3) under the five different conditions indicated (mean ± SEM; n = 20 cells). NO and NP stand for the nucleolus and nucleoplasm, respectively. * *p* < 0.05.

To compare biophysical properties of the nucleolus with other compartments, RI value of the nucleolus, nucleoplasm, and cytoplasm was quantified under varying physiological conditions (Figure 4A). Under normal conditions of DMEM culture, the mean RI of the nucleolus (1.363) was much higher than those of the cytoplasm and nucleoplasm (1.349). It should be noted that the same pseudo-color of cytoplasm and nucleoplasm with a single broad RI band was used for simplicity and comparison between the conditions. Interestingly, each RI value of the compartments was differently changed depending on applied conditions. For the nucleolus, no significant change in RI was detected for hypertonic culture and ATP depletion, but significant change was detected by PBS culture and transcriptional inhibition. In the cytoplasm and nucleoplasm, the RI was changed largely by hypertonic culture, although it was also significantly changed by PBS culture and transcriptional inhibition. The ratios of change in the RI of the nucleolus and cytoplasm (nucleoplasm) under hypertonic conditions were 0.10% and 0.40%, respectively. In contrast, the ratios of change of the nucleolus and cytoplasm (nucleoplasm) for transcriptional inhibition were 0.36% and 0.16%, respectively. These results suggest that the nucleolus is very different from the cytoplasm and nucleoplasm in sensing physiological change of cells. Evaluating the diffusion coefficient, *D*, of probe molecules by FCS is a well-known method for calculating the fluidic viscosity of cellular compartments and the state of molecular crowding in cells as well as aqueous solutions [20,48]. It is noticed that dynamic range of *D* value change is much broader than that of RI value change, as demonstrated in a previous study [34]. As previously demonstrated [21], diffusional mobility of pentameric GFP was evaluated in the nucleolus and nucleoplasm in live HeLa cells under varying physiological conditions (Figure 4B). The average *D* values for pentameric GFP both in the nucleolus and nucleoplasm decreased remarkably under hypertonic culture conditions. However, the ratio of change from normal to hypertonic culture condition for the nucleolus (26%) is much smaller than that for the nucleoplasm (60%). The result is in close agreement with that obtained from RI evaluation. ATP depletion and transcriptional inhibition also largely changed the *D* value in the nucleolus compared with normal conditions, even though the *D* value in the nucleoplasm was only slightly changed by the conditions. These results are consistent with our previous studies [20,21]. In contrast, the *D* value in the nucleolus was not influenced by PBS culture, while the *D* value in the nucleoplasm was slightly increased in PBS culture. The lack of change in the D value of the nucleolus of cells under PBS culture was not consistent with the significant decrease in the RI value in the nucleolus. Nevertheless, the results of FCS also support that the nucleolus is very different from the cytoplasm and nucleoplasm in sensing physiological changes in cells such as hyper-osmotic stress and transcriptional inhibition.

### 3.4. ODT Single Cell Tracing before and after ATP Depletion or Transcriptional Inhibition

**Figure 5:**
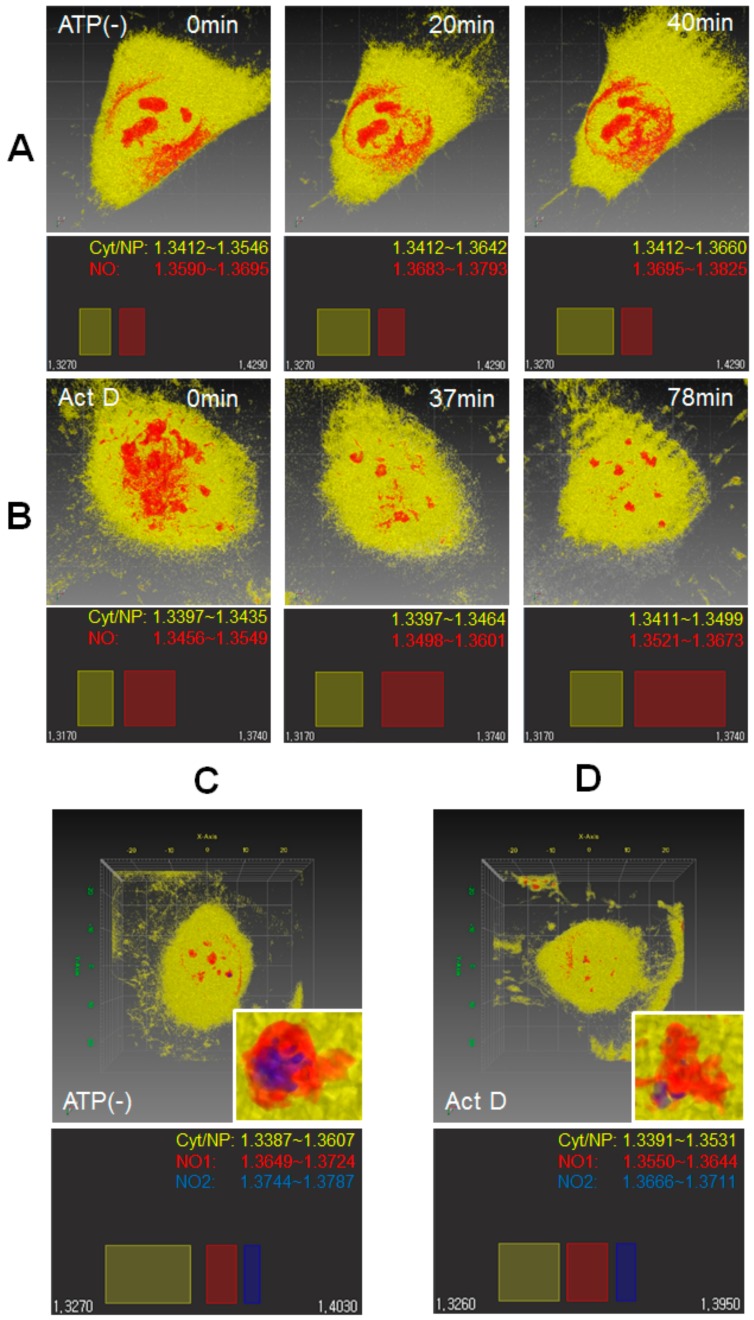
ODT time-lapse analysis of single HeLa cells with ATP depletion or Act D treatment. A single HeLa cell was traced by ODT over a 40 min period following media change for ATP depletion (**A**) and over an 80 min period following addition of Act D (4 μg/mL) (**B**). Representative pseudo-colored RI images of identical HeLa cell during indicated time-lapse intervals (upper panel). In pseudo-colored images, the cytosol and nucleolus are represented by yellow and red, respectively. It should be noted that for structural clarity and comparison between the time intervals, the same pseudo-color of the cytosol and nucleolus was used. Corresponding RI ranges for the cell membrane (white), cytoplasm/nucleoplasm (yellow), and nucleolus (red) are indicated (low panel). (**C**,**D**) HeLa cells with multiple nucleoli are shown after ATP depletion (30 min) and Act D treatment (120 min), respectively (upper panel). Enlarged RI images of a small nucleolus with unique 3D structure are also shown (inset, see also Supplementary Videos S7 and S8). Enlarged RI images of the nucleolus are represented by discontinuous RI bands as indicated (low panel). Cyt, NP, and NO stand for the cytoplasm, nucleoplasm, and nucleolus, respectively. Each range of RI value was indicated. Scale square, 10 × 10 μm.

Since the dynamic range of RI value is narrow (i.e., insensitive to small change in molecular density), ensemble average of different sizes of nucleoli of heterogeneous HeLa cells during cell cycle can miss a small and specific change to RI value in single cells, as shown in Figure 4A. Fortunately, effects of ATP depletion and transcription inhibition on cells occur slowly, ranging from 20 to 80 min, while effects of osmotic stress on cells occur seconds after media change. Therefore, we carried out ODT time-lapse observation of a single HeLa cell after ATP depletion or transcription inhibition to find any small change of nucleolar property during the delayed time (Figure 5). Both ATP depletion and transcription inhibition induced morphological change of cell, and cell volume was decreased with time (Videos S3–S6), which is consistent with the result obtained from ensemble average of cells (Figure S1). Previous studies reported that ATP depletion induced nuclear and shrinkage and chromatin reorganization when HeLa cell is expressing H2B-YFP or H2B-RFP were observed by CLSM [20,46]. For ATP depletion, the RI value of the nucleolus was significantly changed from 1.364 to 1.376 over time, although the morphological changes in the nucleolus were not significant (Figure 5A). In contrast, the RI value of the cytoplasm and nucleoplasm changed slightly from 1.348 to 1.354. This indicates that the ratio of change in RI for the nucleolus (0.90%) was 2-fold larger than that for the cytoplasm and nucleoplasm (0.42%). Moreover, part of the nucleoplasm showed a high RI value, which was significantly redistributed at the perinuclear region of the cytoplasm. This suggests that nuclear compartments excluding the nucleolus and cytosolic compartments in the perinuclear region such as the endoplasmic reticulum and Golgi can be reorganized by ATP depletion. For transcriptional inhibition, the nucleolus was largely disrupted, and the RI value of the nucleolus was significantly increased through time, while RI value for the cytoplasm was little changed into high-RI region (Figure 5B), showing that ratio of change in RI for the nucleolus is 2.4-fold larger than that for the cytoplasm and nucleoplasm. The results of a single-cell time-lapse observation support that the RI value of the nucleolus in the identical cell is largely changed by ATP depletion as well as transcriptional inhibition. Based on the result, we searched the nucleoli of ATP-depleted cells to find the nucleolus, the RI value of which was higher than that of surrounding nucleoli (Figure 5C, Video S7). Interestingly, cells with a small nucleolus and high RI value in ATP-depleted cells were detected, and the nucleolus was represented by two discontinuous RI bands. One of these bands was broad, as shown in Figure 1C and Figure 2A–E, while the other was narrow with a high RI value. Transcriptionally inhibited cells also presented the nucleolus, represented by two discontinuous RI bands (Figure 5D, Video S8). Because of the inverse relationship between RI and *D* values, the increase of RI value in the small part of the nucleolus suggests that the *D* value of the GFP probe decreases in the nucleolar region of high RI. Accordingly, an increase of RI in the nucleolus of ATP depleted and transcriptionally inhibited cells is consistent with a decrease of *D* values in the nucleolus shown in Figure 4B, supporting the results of previous studies [20,21].

Pathologically, it has been indicated that the nucleolus reflects a series of metabolic changes that characterize cancer cells and that the cells in malignant tumors show particularly larger nucleoli compared to normal cells [49,50,51]. This suggests that changes in the physicochemical properties of the nucleolus, such as nucleolar hypertrophy and hyperdensity, can be considered as cytological parameters useful for diagnosing malignancy. Recently, various nuclear bodies, including the nucleolus, have been considered as LLPS droplet organelles, and it has been suggested that maturation of the LLPS into aberrant phase-separated solid aggregates of proteins is closely related to disease and aging such as neurological disorders [52]. Moreover, the nucleolus has functions in several types of cellular stress responses such as DNA damage, transcription-inhibiting stresses, and proteotoxic stress [10]. Thus, the nucleolus as an LLPS organelle serves as an active regulatory site for the detention of extranucleolar proteins related to nucleolar aggresomes or amyloid bodies depending on the stress type and extent of accumulation, although the molecular mechanisms of these processes are unclear. Because of their high molecular density, it is expected that such nucleolar aggresomes or amyloid bodies can be detected by ODT as demonstrated in this study. Although our study combining CLSM, FCS, and ODT analyses focused only on a cancer cell line and several physiological conditions, comparisons of the physicochemical parameters of the nucleoli among various cell types under different physiological conditions will be useful for characterizing and quantifying nucleolar malignancy.

## 4. Conclusions

Our study establishes a new method to quantitatively investigate the physicochemical properties of the nucleoli of live cells by combining label-free ODT 3D imaging with conventional confocal microscopy and highly sensitive FCS analysis. We verified 3D rendered RI images of the nucleolus in HeLa cells by a comparison with 3D confocal images of B23-RFP-expressed cells. Finally, we examined the effect of different physiological conditions on the RIs of the nucleolus and diffusion coefficient of the GFP probe. Our complimentary method demonstrates that biophysical properties of the nucleolus in live cell are sensitive to ATP depletion and transcriptional inhibition, while being insensitive to hyper osmotic pressure when compared with other compartments such as the cytoplasm and nucleoplasm. The result suggests that the nucleolus has unique physicochemical properties when compared with other cellular components. Our approach provides further insight into the correlation between biophysical properties of the nucleolus (i.e., structural sensing of stress) and cellular/nucleolar response under various physiological conditions. Further studies using our method will helpful for diagnosing diseases related to nuclear bodies known as LLPS organelles or revealing the molecular mechanisms governing the spatiotemporal maturation process of LLPS into aberrant phase-separated solid aggregates of proteins in live cells.

## Supplementary Materials

The following are available online at https://www.mdpi.com/2073-4409/8/7/699/s1, Figure S1: Mean value of cell volume for HeLa cells, Video S1: 3D structure of the nucleoli in a live Hela cell represented by single RI band, Video S2: 3D structure of the nucleoli in a live Hela cell represented by three different RI bands, Video S3: Time lapse pseudo-colored RI image of a Hela cell after ATP depletion treatment, Video S4: Time lapse pseudo-colored RI image of a Hela cell after actinomycin D treatment, Video S5: Time lapse raw RI image of a Hela cell after ATP depletion treatment, Video S6: Time lapse raw RI image of a Hela cell after actinomycin D treatment, Video S7: 3D structure of the nucleoli in a Hela cell after ATP depletion treatment, Video S8: 3D structure of the nucleoli in a Hela cell after actinomycin D treatment.
