# Supplementary material for: Physicochemical Properties of Nucleoli in Live Cells Analyzed by Label-Free Optical Diffraction Tomography
Source: Cells. 2019 Jul 10;8(7):699. doi: 10.3390/cells8070699 (PMC6679011; doi:10.3390/cells8070699)
Supplement: Supplementary file 1 [file cells-08-00699-s001.zip › Supp Fig/Supplementary_Material_TKK.docx]

Supplementary Material

# Supplementary Figures

## Supplementary Figures

**Supplementary Figure S1.** Mean value of cell volume for HeLa cells varying physiological conditions is shown. The volumes of HeLa cells were measured by ODT at the five different conditions indicated (mean ± SEM; n = 25 cells).

*****

*****

*****
