# Supplementary figures and images for: Physicochemical Properties of Nucleoli in Live Cells Analyzed by Label-Free Optical Diffraction Tomography
Source: Cells. 2019 Jul 10;8(7):699. doi: 10.3390/cells8070699 (PMC6679011; doi:10.3390/cells8070699)

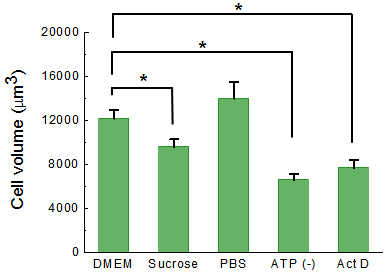

Supplement: Supplementary file 1 [file cells-08-00699-s001.zip › Supp Fig/Figure S1.tif]
